# Etiology of septic arthritis in children of Qatar

**DOI:** 10.1002/emp2.13313

**Published:** 2024-10-17

**Authors:** Abdullah Khan, Abdelmoneem Mohammed Elsheikh, Khalid Alansari

**Affiliations:** ^1^ Department of Emergency Medicine Sidra Medicine Doha Qatar

## Abstract

**Objective:**

Septic arthritis is an orthopedic emergency and if not evaluated and treated appropriately, it can lead to poor clinical outcomes. Previously several studies have been performed to identify the etiology of septic arthritis in the pediatric population in developed countries. The main objective of our study was to identify the etiology of septic arthritis in children in Qatar in previously healthy and fully vaccinated children.

**Methods:**

We performed retrospective chart analysis of children presenting to our emergency department between July 2018 and June 2024, who were diagnosed and treated with septic arthritis. The study was conducted at a level 1 pediatric trauma center and the only children's hospital in the country. We used ICD 9 and ICD 10 codes to identify such cases. After using predefined exclusion criteria, children with positive blood cultures, blood titers for Brucella and/or synovial cultures were included in the analysis. Clinical symptoms and signs, ultrasound findings, and culture results were tabulated using descriptive statistics.

**Results:**

A total of 45 patients were included. The median age of children was 5 years (interquartile range [IQR] 2–10 years). Majority (60%) were male. The most common clinical findings were limping/limitation of joint movement (100%), fever (80%), and swelling of joints (58%). The median C‐reactive protein and erythrocyte sedimentation rate were 94 mg/L and 47 mm/h. The knee and hip were the most common joints affected. The most common causative organisms were *Staphylococcus aureus* (56%), *Streptococcus pyogenes* (13%), and Brucella (11%). Pre‐intervention imaging, such as ultrasound and/or magnetic resonance imaging, was performed in 95% of patients. All patients recovered without any complications. One of the limitations of our study is that cases of *Kingella kingae* septic arthritis may be underreported as polymerase chain reaction (PCR) analysis of synovial fluid was not performed on all patients.

**Conclusion:**

Gram‐positive cocci, especially *S. aureus*, remains the most common cause of septic arthritis in vaccinated children. We also identified Gram‐negative bacilli as causative organisms in our study. We suggest including empiric coverage for both Gram‐ and Gram‐negative bacilli when treating children with suspected septic arthritis till culture results are available.

## 1 INTRODUCTION

### 1.1 Background

Acute septic arthritis in children is a clinical emergency. It is caused by hematogenous invasion of synovial lining by microorganisms, spread from a nearby infection or direct inoculations by a trauma. Once inside the joint, the microorganisms proliferate and trigger an immune response causing rapid destruction of the joint.^1^ If not treated promptly, septic arthritis is associated with increased intracapsular pressure leading to ischemia, destruction of cartilage, and osteonecrosis. The incidence of septic arthritis varies across the globe with an incidence of 4/100,000 children in the United States, 4–35/100,000 in Australia, and 2–12/100,000 children in the Middle east.^2^ Septic arthritis is commonly a monoarticular infection in children. Hip and knee are the commonly affected joints.

### 1.2 Importance

The etiology of septic arthritis depends on multiple factors such as age, history of vaccination, immunosuppression, and location.  Previously, *Haemophilus influenzae* and *Streptococcus pneumoniae* were common causes of invasive diseases and septic arthritis.^3^, ^4^ After the introduction of childhood vaccines, there has been a decline in the incidence of septic arthritis caused by these organisms and emergence of *Staphylococcus aureus*, *Kingella kingae*, and *Streptococcus pyogenes* as common causative organisms.^5^, ^6^ On the contrary, salmonella infection is responsible for 40%–60% of the cases of septic arthritis in children of sub‐Saharan African countries.^7^ Since microorganisms causing septic arthritis can vary across the globe, it is important identify the common microorganisms in different populations. This will guide the choice of initial antibiotics till culture results are ready.

### 1.3 Goals

Qatar spent significant efforts in implementing a childhood vaccination program over the last few decades. As a result, Qatar has surpassed the global benchmark of childhood vaccination coverage.^8^ The main purpose of our study was to identify the etiology of septic arthritis in previously healthy, non‐immunosuppressed and fully vaccinated children of Qatar. To our knowledge, this is the first study conducted in Qatar to identify the etiology of septic arthritis in children.

## 2 METHODS

### 2.1 Study design

This is a retrospective observational study of all patients (ages 0 and 18 years) who presented to our emergency department from July 1, 2018 to June 30, 2024, and were diagnosed as septic arthritis. The study was approved by the institutional review board (IRB 210784).

### 2.2 Setting

Qatar is a country located in the Middle east with an estimated population of 3 million. The study was conducted at the pediatric emergency department of Sidra Medicine. Sidra Medicine is the only dedicated tertiary children hospital and level 1 pediatric trauma center in the country with an estimated 100,000 pediatric visits annually. All children requiring a higher level of care and specialty consultation, such as pediatric orthopedics (for suspected septic joints and fractures), are referred to us from across the country. Therefore, subjects in our study are a valid representation of children in Qatar.

**The Bottom Line**

In our study to identify the etiology of septic arthritis in children of Qatar, we observed both Gram‐positive cocci and Gram‐negative rods as causative organisms. Therefore, it is important to empirically treat cases of septic arthritis with antibiotics targeting both groups till culture results are available.

### 2.3 Selection of subjects

We identified eligible patients for our study by using the international classification of diseases (ICD‐9 and ICD‐10) diagnostic codes for septic arthritis. For our study, we defined a *positive evaluation for septic arthritis* as the presence of positive synovial fluid Gram stains or cultures, positive blood titers for Brucella (>1:160), and positive blood cultures for pathogens. A *negative evaluation for septic arthritis* was defined as negative cultures (blood and synovial) and blood titers for Brucella. We used these definitions with slight modification (addition of positive blood titers for Brucella) from the widely cited study on pediatric septic arthritis by Young et al.^6^

We excluded patients based on the following exclusion criteria: duplicate visits, history of chronic medical conditions, history of infection or surgery in the affected joint, positive blood cultures with organisms considered as contaminants, and negative evaluation for septic arthritis (as defined above). Patients were also excluded if alternative diagnosis was made without synovial fluid evaluation. Chronic medical condition was defined as the presence of chronic cardiac, pulmonary, renal, gastrointestinal, hepatic, hematological (leukemias and blood dyscrasias), or neurological conditions; chromosomal abnormalities; and carcinomas.

### 2.4 Measures and outcomes

The primary outcome of interest was microbiological etiology of septic arthritis. We used a standardized form for data collection. Data were collected by a fellow in‐training physician and later was verified by a pediatric emergency attending physician. The physicians were not blinded to the study objectives. There was no disagreement among the involved physicians regarding eligible patients, study design, data collection (measures and outcome), analysis, and results.

The data related to patient demographics, clinical signs and symptoms, type of joint involved, radiological studies, synovial fluid analysis (synovial fluid white cell count and percentage of neutrophils) and synovial fluid cultures, peripheral blood analysis (white blood count [WBC], C‐reactive protein [CRP], and erythrocyte sedimentation rate [ESR]), blood titers for Brucella, and peripheral blood cultures were extracted from the electronic health records. Limping and limitation of joint movement were counted as single clinical variables to account for upper extremity joints.

### 2.5 Data analysis

We used descriptive statistics for the analysis of the data. For categorical variables, proportions and 95% confidence interval (CI) were reported using modified Wald technique. For continuous variables, median and interquartile range (IQR) were reported. The SPSS software (IBM Corp) was used for data analysis.

## 3 RESULTS

**FIGURE 1:**
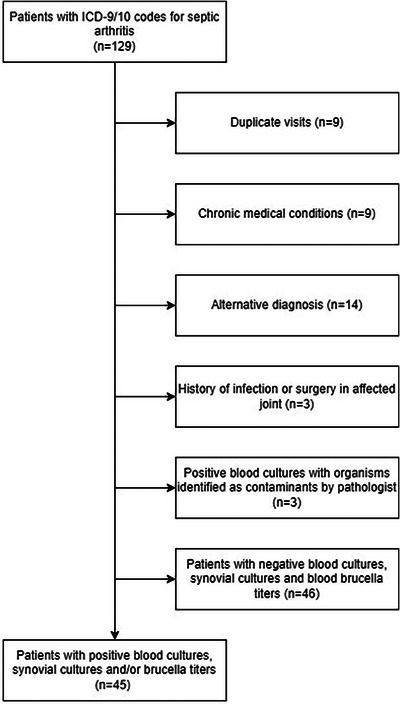
Patient flow diagram.

We identified a total of 129 patient encounters using ICD 9 and 10 diagnostic codes for septic arthritis. Nine duplicate patient encounters were excluded. Nine patients with chronic medical conditions (one patient each had chromosomal abnormality, nephrotic syndrome, chronic kidney disease with obstructive uropathy, end stage renal disease, tethered cord syndrome, acute myeloid leukemia, renal cell carcinoma, and two patients had seizure disorder) were excluded. Fourteen patients were excluded based on alternative diagnosis and did not get synovial fluid analysis. Out of these, 11 patients were diagnosed as post infectious arthritis, one patient each with trochanteric bursitis, tenosynovitis, and rheumatic fever. Three patients with positive blood cultures secondary to organisms considered as contaminants were also excluded. *Staphylococcus capitis*, *Brevibacterium casei*, and viridian group streptococci were identified in these patients. Three patients with a previous history of septic arthritis in the same joint were also excluded. An additional 46 patients with negative evaluation for septic arthritis were also excluded. A total of 45 patients with positive evaluation for septic arthritis were included in the final analysis. Each patient had a single joint affected (Figure 1).

The median age was 5 years (IQR, 2–10 years). Majority of patients (60%) were males. The most common signs and symptoms were limping/limitation of joint movement, fever, and swelling of the joint (Table 1). Among patients with hip and shoulder septic arthritis, only one patient had clinically noticeable joint swelling. Hip and knee were the most affected joints. The median WBC was 12 × 10^9^/L and median CRP and ESR were 94 mg/L and 47 mm/h, respectively. Ultrasound (US) and/or magnetic resonance imaging (MRI) was performed in 43 (95%) patients before the joint aspiration. Either US (27 patients) or MRI (10 patients) was performed in 35 patients; whereas, US was followed by MRI in eight patients. On US, joint effusion was identified in all patients with a median depth of effusion of 7.15 mm. Synovial thickening was noticed in 69% of the patients. On MRI, joint effusion and synovial enhancement was noticed in all patients.

**TABLE 1:** Clinical features of patients.

Clinical characteristics	Number (frequency); confidence interval (*n* [%]; 95% CI, %), median; interquartile range (IQR)^a^
**Symptoms and signs**	
Fever	36 (80%); 65–90
Temperature^a^(°C)	38.3; 37.7–38.8
Swelling of joint	26 (58%); 42–72
Limping/limitation in joint movement	45 (100%)
Warm to touch	20 (44%); 30–60
Erythema	9 (20%); 10–35
Duration of symptoms (days)	3; 2–5
**Joints affected**	
Hip	18 (40%); 26–56
Knee	16 (36%); 22–51
Ankle	7 (16%); 6–29
Shoulder	2 (4%); 0–15
Elbow	2 (4%); 0–15
**Pre‐intervention ultrasound (US) of the joint**	
US joint	35 (78%)
Joint effusion	35 (100%)
Joint effusion depth (mm)^a^	7.15 (4.5–20)
Synovial thickening	24 (69%); 51–83
Synovial erythema	9 (26%); 12–43
**Blood analysis**	
White blood count (count ×10^9^ /L)^a^	12; 8.3–16
Absolute neutrophil count (count ×10^9^ /L)^a^	6; 4.2–9.5
Erythrocyte sedimentation rate (mm/h)^a^	47; 33–85
C‐ reactive protein (mg/L)^a^	94; 50–137
**Synovial fluid analysis**	
Synovial fluid appearance	
Purulent	23 (51%); 36–66
Turbid	18 (40%); 26–56
Bloody	2 (4%); 0–15
Clear	2 (4%); 0–15
Synovial fluid WBC (count/mm^3^)^a^	32750; 6750–87,900
Synovial fluid ANC percentage	86; (74–95)
**Positive culture results**	
Synovial fluid cultures	29 (64%); 49–78
Blood cultures/Brucella titers	7 (16%); 6–29
Both synovial and blood cultures/Brucella titers	9 (20%); 10–35

Abbreviations: ANC, absolute neutrophil count; C, degree celsius; mm/h, millimeter per hour; mg/L, milligram per liter; mm^3^, cubic millimeter.

^a^ Continuous variables described as median and interquartile range.

The joint aspiration was performed on all patients. A sample for synovial fluid cell count was sent to 24 (53%) patients with median synovial fluid WBC of 32,750/mm^3^. The blood and/or synovial cultures were positive in all patients (Table 1). The most common organisms were Gram‐positive cocci identified in 34 (76%) patients. The methicillin‐sensitive *S. aureus* and community‐acquired methicillin‐resistant *S. aureus* (CA‐MRSA) were the predominant Gram‐positive cocci noted in 25 (56%) patients. Five CA‐MRSA isolates were susceptible to clindamycin, whereas three isolates were resistant to clindamycin but sensitive to vancomycin. The Gram‐negative rods were identified in 11 (24%) patients. Brucella was the most common Gram‐negative rod (Table 2).

**TABLE 2:** Common causative organisms.

Microorganisms isolated	*n* (%; 95% CI, %)
**Gram‐positive cocci**	34 (76; 60–87)
Methicillin‐sensitive *Staphylococcus aureus*	17 (38; 24–53)
CA‐MRSA	8 (18; 8–32)
*Streptococcus pyogenes*	6 (13; 5–27)
*Streptococcus pneumonia*	2 (4; 0–15)
*Streptococcus dysglactasia*	1 (2; 0–12)
**Gram‐negative rods**	11 (24; 13–40)
Brucella	5 (11; 4–24)
Salmonella	2 (4; 0–15)
*Kingella kingae*	2 (4; 0–15)
*Escherichia coli*	1 (2; 0–12)
Hemophilus influenza	1 (2; 0–12)

Abbreviations: ANC, absolute neutrophil count; CA‐MRSA, community‐acquired methicillin‐resistant *Staphylococcus aureus*; CI, confidence interval.

### 3.1 Limitations

Our study reports on a small number of children. Our laboratory does not test for specific serotypes of *H. influenzae* and streptococcus pneumonia. Therefore, we were not able to determine if cases of septic arthritis caused by these organisms are secondary to vaccine preventable serotypes. Similarly, we were unable to identify the true number of cases of *K. kingae* because PCR analysis to detect *K. kingae* was not performed in the majority of culture negative septic arthritis.

## 4 DISCUSSION

The etiology of septic arthritis in children has changed after the introduction of childhood vaccination programs across the world. Our study indicates that *S. aureus* was the most common cause of septic arthritis accounting for half of the cases. Approximately one‐fourth of the cases of septic arthritis were caused by Gram‐negative rods. Brucella was the most common Gram‐negative rod identified in our population. Hip and knee were the commonly affected joints followed by ankle, shoulder, and elbow. Elevated ESR (>20 mg/L) and CRP (>20 mm/h) were noted in most of the patients.

Previously studies done in western children have reported staphylococcus as a predominant cause of septic arthritis in 44%–63% of the cases.^6^, ^9^, ^10^ Similarly, a study done in children of Saudia Arabia noted that 39% of the cases were caused by *S. aureus*.^11^ In the last two decades, the prevalence of osteoarticular infections caused by CA‐MRSA have changed variably in different populations. In a study by Arnold et al., the proportions of CA‐ MRSA infection increased from 4% to 40% in their population.^12^ However, in a study by Weiss et al, the prevalence of CA‐MRSA‐related osteoarticular infections decreased significantly from 45% to 18%.^13^ In our study, 18% of cases of septic arthritis were caused by CA‐MRSA, which is comparable to the number of cases observed by Jain et al.^14^ We observed *S. pyogenes* in 13% of our cases, which is similar to the reports by Calvo et al. (9%), Moumile et al (13.5%), and Peltola et al (12%).^9^, ^10^, ^15^ The incidence of *Streptococcus pneumoniae* septic arthritis is reported between 1% and 7% in different studies.^5^, ^16^ We identified two patients (4%) with streptococcal pneumonia septic arthritis. We also observed a case of septic arthritis caused by *Streptococcus dysgalactiae*, which recovered without any complications. Although rare, case reports of pediatric arthritis caused by this organism  have been reported with good clinical outcomes.^17^, ^18^ Among Gram‐negative rods, Brucella was the most common organism, noted in four cases (11%). Brucella is a common zoonotic infection in middle east acquired after consumption of raw milk or contact with animals infected with Brucella.^19^ Leukopenia, thrombocytopenia, and transaminitis are prominent laboratory findings in Brucella osteoarticular infection.^20^, ^21^ None of these findings were noticed in our cases. Osteoarticular infections secondary to salmonella are common in children with immunosuppression, neutropenia, malnutrition, sickle cell anemia, or habitat in areas where salmonella infection is endemic.^22^ Although rare, Pezone et al and Tirta et al have reported cases of salmonella septic arthritis in immunocompetent children.^23^, ^24^ In our study, salmonella was identified in two (4%) children and both children had no clinical or laboratory evidence of immunological deficiencies or blood dyscrasias. *Escherichia coli* is an uncommon cause (incidence less than 1%) of septic arthritis, commonly noted in neonates with prematurity.^5^, ^25^, ^26^ We observed a single case of *E. coli* septic arthritis in a 3‐week‐old neonate, born full term with no risk factors for sepsis.

In children with suspected septic arthritis, it is suggested to aspirate the joint for synovial fluid analysis, if CRP and ESR are >20 mg/L and 20 mm/h.^1^ In our study, 41 of 44 patients (93%) had CRP >20 mg/L with a median of 94 mg/L and 36 of 40 patients (90%) had ESR greater than 20 mm/h with median of 47 mm/h, which are similar to the results published by Pääkkönen M et al^27^ Regarding synovial fluid analysis, synovial leucocyte count greater than 50,000/mm^3^ with predominance of neutrophils (>75%) is suggestive of septic arthritis.^1^ Aupiais et al reported  a median synovial fluid leukocyte count of 147,000/mm^3^ in children with septic arthritis.^28^ Interestingly, in our study, median synovial WBC was less than 50,000/mm^3^ (32,750/mm^3^), which can be due to the small number of synovial samples (*n* = 24) analyzed for synovial WBC. Only, 42% (9/24) of the patients had synovial WBC greater than 50,000/mm^3^, which is also lower than reported by Thomas et al.^29^ In our study, US of the affected joint noted effusion in all patients with median depth of effusion of 7.15 mm similar to previously reported in previous studies.^30^

The interesting finding in our study is the identification of a wide variety of microorganisms as a cause of pediatric septic arthritis. Although children were fully vaccinated, strep pneumonia was still identified as a causative organism. This can be explained by the fact that in Qatar, the pneumococcal vaccines have low coverage rates against the prevalent serotypes especially in younger children.^31^ Although the incidence of invasive disease due to *H. influenzae* (type B) has significantly dropped after the introduction of the vaccine, other serotypes of *H. influenzae* (non‐type B) have emerged as a cause of invasive infection in children.^32^ However, *K. kingae* is common in preschool children, only two cases were identified in our study. This can be explained by the fact that *K. kingae* is more commonly detected by PCR than routine cultures and PCR analysis of synovial fluid was rarely performed in our cases.^33^ Brucellosis is common in the middle eastern and Mediterranean region manifesting as fever, hepatosplenomegaly, and arthritis. The incidence of osteoarticular involvement ranges from 13% to 74% in children with brucellosis.^34^ This highlights the need to regularly screen for Brucella in children with septic arthritis in Qatar. The diagnosis of septic arthritis can be challenging in children. A combination of clinical, laboratory (WBC, CRP, ESR, and synovial analysis), and radiological findings (US and MRI) can be helpful to support the diagnosis. Bisht et al noted that CRP >20 mg/L was a strong independent predictor of both hip and knee septic arthritis and ESR >40 mm/h was a strong predictor of hip septic arthritis.^35^ Traditionally, synovial leucocyte counts greater than 50,000/mm^3^ with predominance of neutrophils (>75%) is suggestive of septic arthritis, but based on our findings it is entirely possible to have septic arthritis with synovial WBC below the above‐mentioned cutoff. US is usually the first radiologic study in evaluating septic arthritis, but it cannot differentiate between septic arthritis and transient synovitis and needs to be followed by MRI and aspiration of joints for synovial fluid analysis.^30^

Our study suggests that most cases of septic arthritis in children are caused by *S. aureus*. Our data also highlight Gram‐negative bacilli as a cause of septic arthritis in previously healthy children. Based on our results, we suggest antibiotic coverage for both Gram‐positive cocci and Gram‐negative bacilli when evaluating and treating cases of suspected pediatric septic arthritis, until blood titers/culture and synovial culture results identify specific organisms.

## CONFLICT OF INTEREST STATEMENT

The authors declare no conflicts of interest.
